# MGAT2 deficiency ameliorates high-fat diet-induced obesity and insulin resistance by inhibiting intestinal fat absorption in mice

**DOI:** 10.1186/1476-511X-11-75

**Published:** 2012-06-14

**Authors:** Takuma Tsuchida, Sayaka Fukuda, Hisanori Aoyama, Nobuhiko Taniuchi, Tomomi Ishihara, Noriko Ohashi, Hiroko Sato, Koji Wakimoto, Masaharu Shiotani, Akira Oku

**Affiliations:** 1 Department I, Pharmacology Research Laboratories II, Research Division, Mitsubishi Tanabe Pharma Corporation, 2-2-50, Kawagishi, Toda-shi, Saitama, 335-8505, Japan; 2 Discovery molecular pharmacology Department, Discovery Screening Center, Research Division, Mitsubishi Tanabe Pharma Corporation, Saitama, Japan; 3 Safety Department II, Safety Research Laboratory, Research Division, Mitsubishi Tanabe Pharma Corporation, Chiba, Japan; 4 Target Discovery and Biomarker Research Department, Advanced Medical Research Laboratories, Research Division, Mitsubishi Tanabe Pharma Corporation, Yokohama, Japan

**Keywords:** Acyl-coenzyme A:monoacylglycerol acyltransferase (MGAT), Obesity, Insulin resistance, Triglyceride, Enterocyte, Fatty acid oxidation

## Abstract

**Background:**

Resynthesis of triglycerides in enterocytes of the small intestine plays a critical role in the absorption of dietary fat. Acyl-CoA:monoacylglycerol acyltransferase-2 (MGAT2) is highly expressed in the small intestine and catalyzes the synthesis of diacylglycerol from monoacylglycerol and acyl-CoA. To determine the physiological importance of MGAT2 in metabolic disorders and lipid metabolism in the small intestine, we constructed and analyzed *Mgat2-*deficient mice.

**Results:**

In oral fat tolerance test (OFTT), *Mgat2*-deficient mice absorbed less fat into the circulation. When maintained on a high-fat diet (HFD), *Mgat2*-deficient mice were protected from HFD-induced obesity and insulin resistance. Heterozygote (*Mgat2*^*+/−*^) mice had an intermediate phenotype between *Mgat2*^*+/+*^ and *Mgat2*^*−/−*^ and were partially protected from metabolic disorders. Despite of a decrease in fat absorption in the *Mgat2*-deficient mice, lipid levels in the feces and small intestine were comparable among the genotypes. Oxygen consumption was increased in the *Mgat2*-deficient mice when maintained on an HFD. A prominent upregulation of the genes involved in fatty acid oxidation was observed in the duodenum but not in the liver of the *Mgat2*-deficient mice.

**Conclusion:**

These results suggest that MGAT2 has a pivotal role in lipid metabolism in the small intestine, and the inhibition of MGAT2 activity may be a promising strategy for the treatment of obesity-related metabolic disorders.

## Background

Intestinal fat absorption involves hydrolysis of dietary triglycerides to 2-monoacylglycerol and fatty acids in the lumen by pancreatic lipase [1]. These hydrolysis products are taken up by enterocytes, and triglycerides are resynthesized through the monoacylglycerol pathway, which is catalyzed by acyl-CoA:monoacylglycerol acyltransferase (MGAT) and acyl-CoA:diacylglycerol acyltransferase (DGAT). Another pathway involved in triglyceride synthesis is the glycerol 3-phosphate pathway, a *de novo* pathway that is present in most tissues [2]. In the small intestinal mucosa, the monoacylglycerol pathway accounts for 70–80% of triglyceride resynthesis after a meal because of the large amount of 2-monoacylglycerol released from dietary fat [3-5]. The newly formed triglycerides are then incorporated into chylomicrons with other lipids for secretion into the blood and transport to other tissues such as the liver and adipose tissue.

MGAT acylates monoacylglycerol to yield diacylglycerol. Three isoforms of MGAT enzymes, MGAT1, MGAT2, and MGAT3, have been identified thus far [6-9]. MGAT1 is mainly expressed in the stomach and kidney and expressed at lower levels in adipose tissue and the liver, but is absent in the small intestine [9]. MGAT2 and MGAT3 are highly expressed in the small intestine [6-8,10]. MGAT2 is expressed in both humans and rodents, and the MGAT3 gene is a pseudogene in mice [8,11].

Recently, it was reported that mice lacking *Mgat2* are protected from metabolic disorders induced by high-fat feeding and show increased energy expenditure, suggesting that MGAT2 may be a key determinant of fat absorption and energy metabolism [12]. However, the exact mechanisms of increased oxygen consumption in *Mgat2*-deficient mice are still unknown [12].

In this study, we constructed and analyzed *Mgat2*-deficient mice to examine the physiological importance of MGAT2 in detail, with a focus on lipid metabolism in the small intestine. We included the heterozygote (*Mgat2*^*+/−*^) mice because there were limited data on the phenotype of *Mgat2*^*+/−*^ mice [12].

## Results

### Generation of *Mgat2*-deficient mice

**Figure 1:**
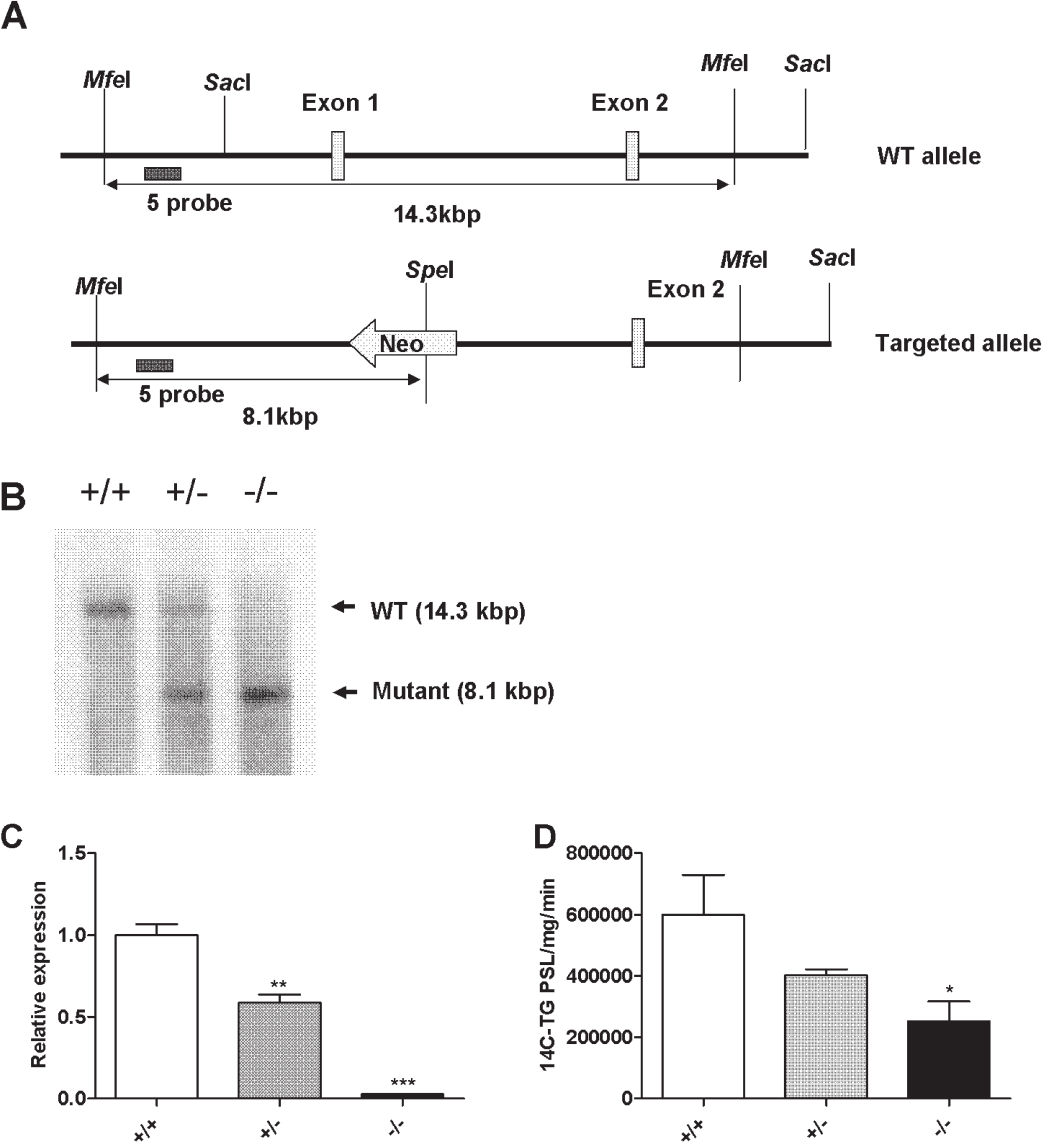
**Generation of*Mgat2*-deficient mice. (A)** Schematic diagram of the derivation of *Mgat2*-deficient mice by homologous recombination. **(B)** Southern blotting of genomic DNA. **(C)** mRNA levels of *Mgat2* in the duodenum. **(D)** Intestinal MGAT activity. Each bar represents mean ± SEM. n = 3–5 per group. ^*^P < 0.05, ^**^P < 0.01, ^***^P < 0.001 vs. +/+.

We generated *Mgat2*-deficient mice as described in Methods (Figure 1A). *Mgat2*^*−/−*^ mice were viable and fertile without apparent abnormalities. *Mgat2* deficiency was confirmed by Southern blotting of genomic DNA (Figure 1B). Gene expression levels of *Mgat2* were reduced in the duodenum of *Mgat2*^*+/−*^ mice and undetectable in *Mgat2*^*−/−*^ mice (Figure 1C). MGAT activity in the total small intestine was reduced in the *Mgat2*-deficient mice (Figure 1D).

### Reduced fat absorption in the *Mgat2*-deficient mice

**Figure 2:**
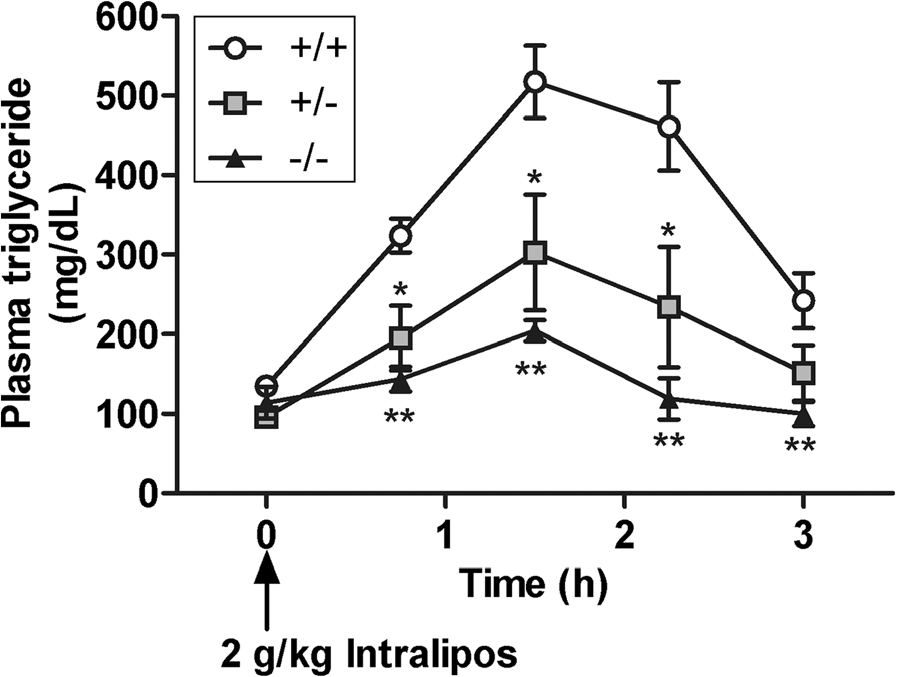
**Reduced fat absorption in the*Mgat2*deficient mice.** Time course of changes in plasma triglyceride levels after an oral challenge of Intralipos. Each bar represents mean ± SEM. n = 5–6 per group. ^*^P < 0.05, ^**^P < 0.01 vs. +/+.

In oral fat tolerance test (OFTT), plasma triglyceride levels were transiently increased in *Mgat2*^*+/+*^ but not in *Mgat2*^*−/−*^ mice (Figure 2), indicating inhibition of triglyceride absorption into *Mgat2*^*−/−*^ mice circulation.

### Reduced body weight gain and adiposity in the *Mgat2*-deficient mice

**Figure 3:**
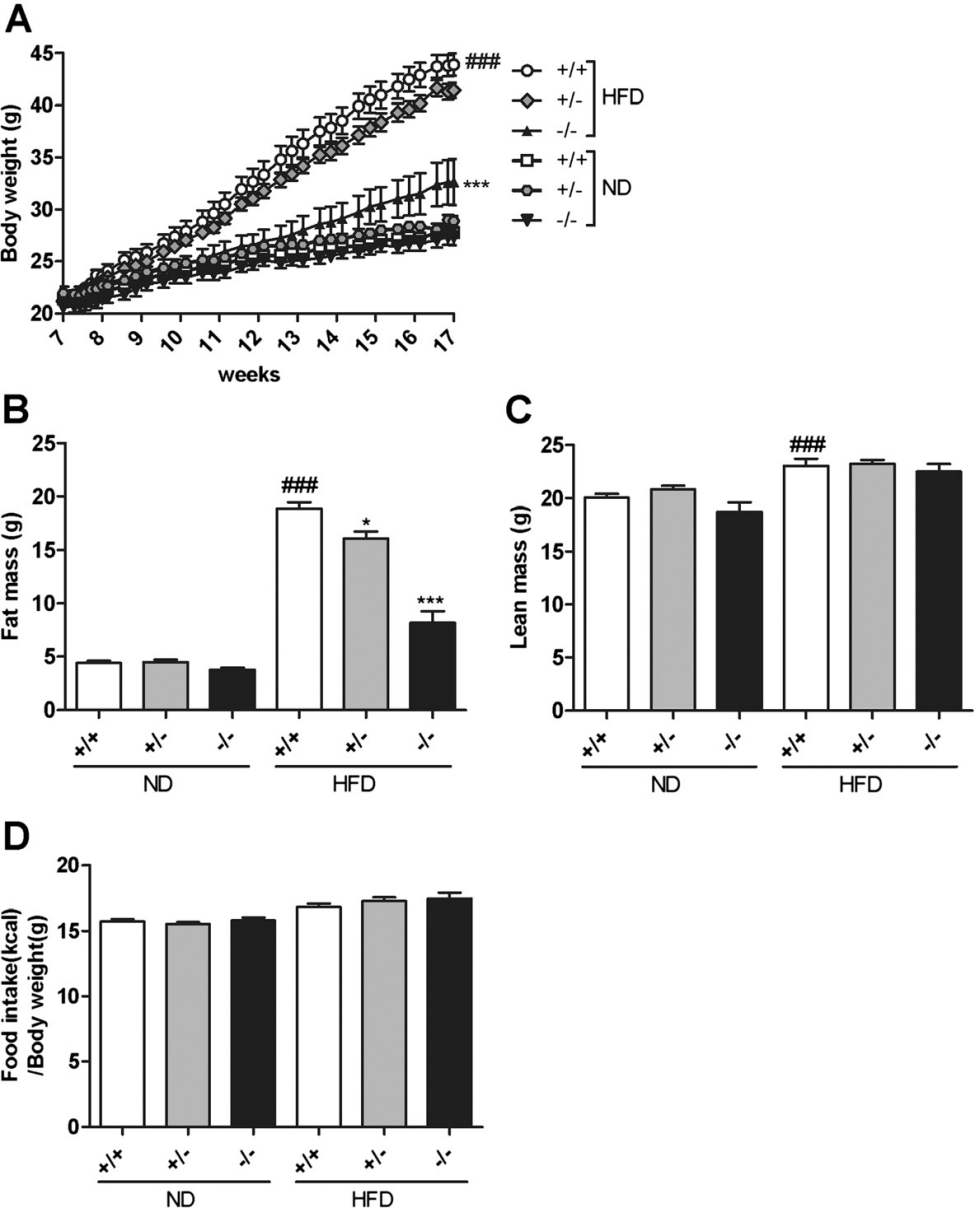
**Reduced body weight gain and adiposity in the*Mgat2*-deficient mice on an HFD.** Time course of changes in body weights **(A)**, difference in fat mass **(B)**, lean mass **(C)**, and total food intake **(D)** after 10 weeks of ND or HFD feeding. Each bar represents mean ± SEM. n = 6–15 per group. ^*^P < 0.05, ^***^P < 0.001 vs. HFD +/+. ^###^*P* < 0.001 vs. ND +/+.

In order to examine the physiological significance of the inhibition of fat absorption by *Mgat2* deficiency on long-term body weight homeostasis, we challenged the *Mgat2*-deficient mice with an ND or HFD. On an ND, *Mgat2*^*+/+*^, *Mgat2*^*+/−*^, and *Mgat2*^*−/−*^ mice showed similar body weight gains (Figure 3A). In contrast, when maintained on an HFD, *Mgat2*^*−/−*^ mice exhibited a markedly reduced rate of body weight gain (Figure 3A). *Mgat2*^*−/−*^ mice showed approximately 26% lower body weight compared with *Mgat2*^*+/+*^ mice after 10 weeks of HFD feeding. *Mgat2*^*+/−*^ mice had an intermediate phenotype. Analysis of body composition showed that fat mass was reduced by approximately 15% in *Mgat2*^*+/−*^ and 57% in *Mgat2*^*−/−*^ mice (Figure 3B), with no difference in lean body mass when maintained on an HFD (Figure 3C). There was no difference in total food intake based on body weight (Figure 3D). Therefore, the *Mgat2*-deficient mice were protected from HFD-induced obesity independent of food intake.

### Improved insulin sensitivity in the *Mgat2*-deficient mice

**Figure 4:**
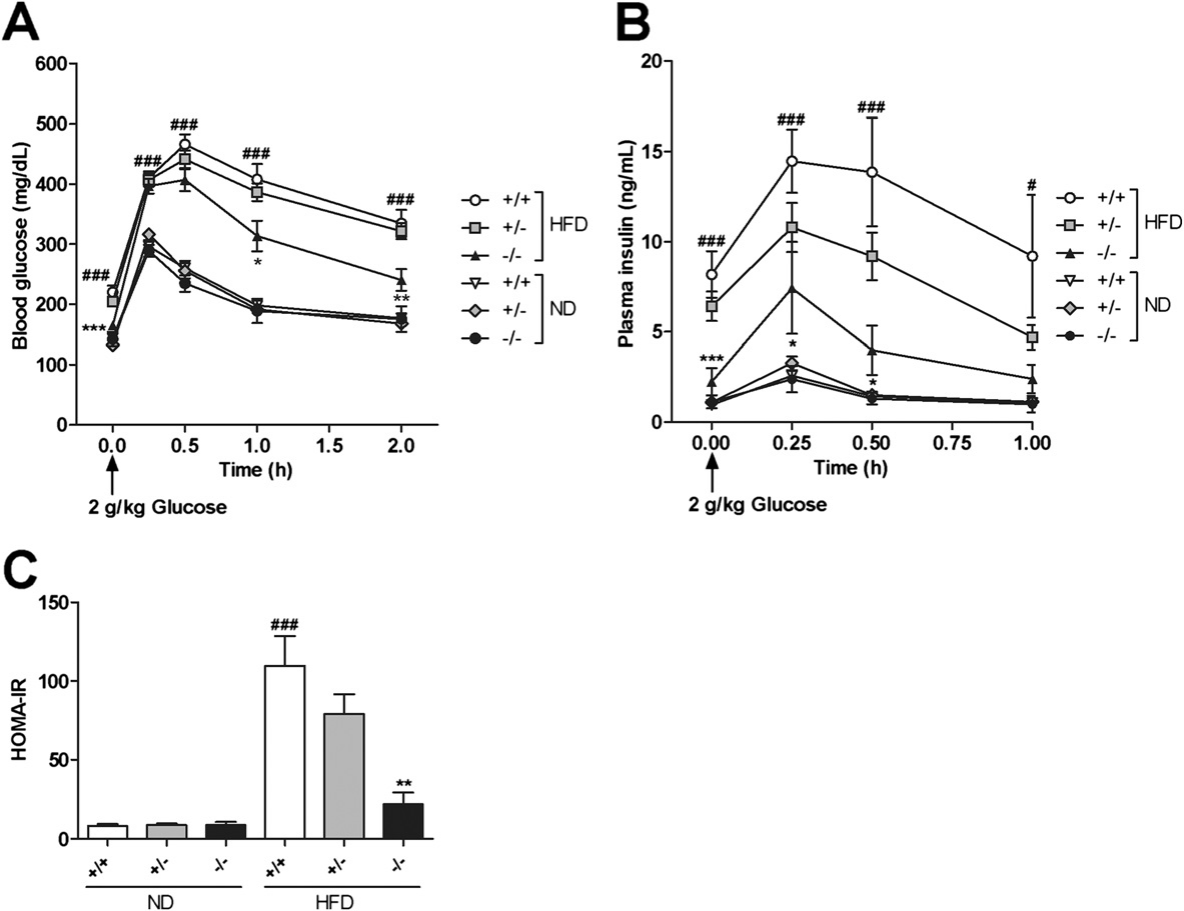
**Improved glucose tolerance and insulin sensitivity in the*Mgat2*-deficient mice on an HFD.** Time course of changes in blood glucose **(A)** and plasma insulin **(B)** after an oral challenge of 2 g/kg glucose. **(C)** HOMA-IR was calculated. Each bar represents mean ± SEM. n = 6–15 per group. ^*^P < 0.05, ^**^P < 0.01, ^***^P < 0.001 vs. HFD +/+. ^#^*P* < 0.05, ^###^*P* < 0.001 vs. ND +/+.

No difference was observed among genotypes in plasma parameters, including blood glucose, insulin, total cholesterol, NEFA, and triglycerides in the fed state (Table 1), and in glucose tolerance (Figure 4A, B) on an ND. When maintained on an HFD, *Mgat2*^*+/+*^ mice showed an expected increase in blood glucose, insulin, and total cholesterol levels (Table 1) as well as in glucose intolerance (Figure 4A, B). However, insulin and total cholesterol levels were significantly reduced in *Mgat2*^*+/−*^ and *Mgat2*^*−/−*^ mice (Table 1). Furthermore, *Mgat2*^*−/−*^ mice exhibited improvement of glucose tolerance (Figure 4A, B) and increased insulin sensitivity as assessed by homeostasis model assessment for insulin resistance (HOMA-IR) (Figure 4C). These data suggested that the *Mgat2-*deficient mice were protected from HFD-induced insulin resistance and hypercholesterolemia. Subsequently, we mainly focused on the HFD-fed groups because no change was observed in the plasma parameters and body weights of the ND-fed groups.

**Table 1:** **Plasma parameters in the fed state of the** ***Mgat2*-deficient mice**

	ND			HFD		
Parameter	*Mgat2*^+/+^	*Mgat2*^+/−^	*Mgat2*^−/−^	*Mgat2*^+/+^	*Mgat2*^+/−^	*Mgat2*^−/−^
Glucose (mg/dL)	132 ± 4	130 ± 3	135 ± 5	168 ± 6^###^	168 ± 5	151 ± 7
Insulin (ng/mL)	1.4 ± 0.2	1.7 ± 0.4	2.0 ± 1.0	35.8 ± 10.4^##^	14.6 ± 3.0^*^	5.7 ± 1.8^*^
Triglyceride (mg/dL)	114.5 ± 12.5	123.2 ± 12.7	90.5 ± 5.6	102.7 ± 6.5	119.3 ± 7.4	94.4 ± 7.9
Total cholesterol (mg/dL)	81 ± 5	89 ± 4	94 ± 7	196 ± 14^###^	137 ± 6^***^	118 ± 6^***^
NEFA (mEq/L)	0.45 ± 0.03	0.43 ± 0.04	0.36 ± 0.04	0.46 ± 0.02	0.45 ± 0.02	0.46 ± 0.03

Data are expressed as mean ± SEM. n = 6–15 per group. ^*^P < 0.05, ^***^P < 0.001 vs. HFD +/+. ^##^*P* < 0.01, ^###^*P* < 0.001 vs. ND +/+.

### Higher oxygen consumption in the *Mgat2*-deficient mice

**Figure 5:**
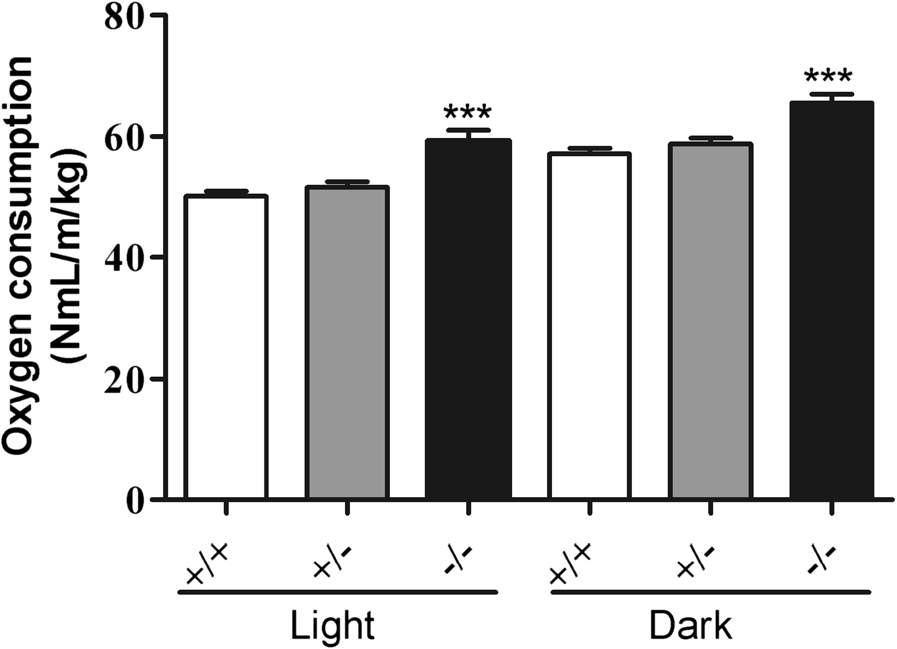
**Increased oxygen consumption in the*Mgat2*-deficient mice.** Average oxygen consumption during light–dark phase was measured. Each bar represents mean ± SEM. n = 6–15 per group. ^***^P < 0.001 vs. HFD +/+.

Reduced body weight gain without alteration in food intake in the *Mgat2*-deficient mice suggested alterations in energy metabolism. Oxygen consumption during the light–dark phase was significantly higher in *Mgat2*^*−/−*^ mice on an HFD (Figure 5).

### Lipid levels in feces and the small intestine

**Figure 6:**
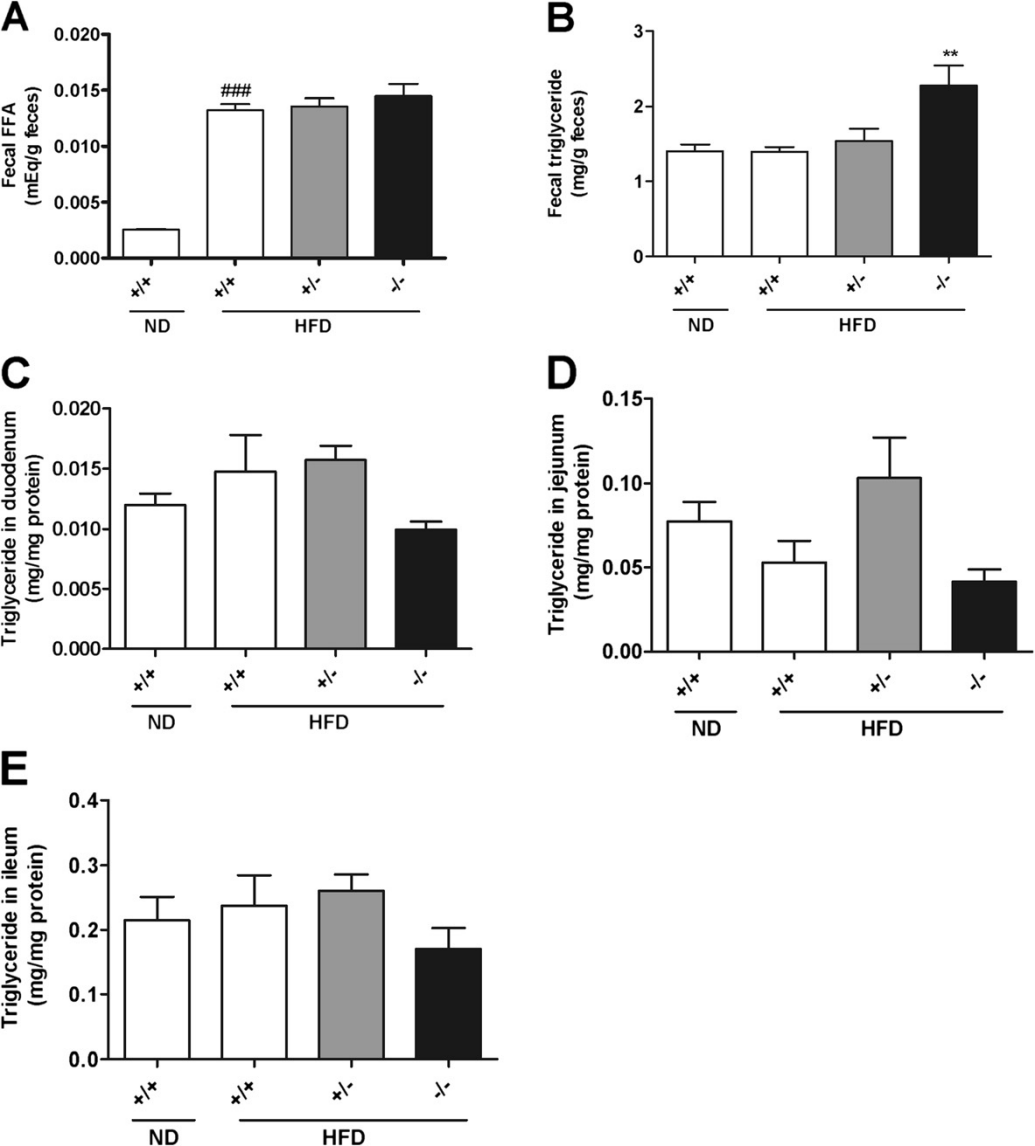
**Lipid levels in the feces and small intestine.** Fecal levels of NEFA (**A**) and triglycerides (**B**), and triglyceride levels in the duodenum (**C**), ileum (**D**), and jejunum (**E**) were measured. Each bar represents mean ± SEM. n = 3–15 per group. ^**^P < 0.01, vs. HFD +/+. ^###^*P* < 0.001 vs. ND +/+.

We measured lipid levels in feces and the small intestine to investigate the metabolic fate of unabsorbed triglycerides as a result of *Mgat2* deficiency. NEFA levels in feces was similar among genotypes (Figure 6A), and triglyceride levels in the feces were slightly increased (Figure 6B). However, fecal outputs of triglycerides were less than 1% of those contained in an HFD, indicating that the majority of ingested fat exhibited normal uptake by the enterocytes in the *Mgat2*-deficient mice. Triglyceride levels in the duodenum, jejunum, and ileum were not different among genotypes (Figure 6C–E). Histological examination also showed no difference in the amount of lipid droplets in the duodenum (data not shown). These results suggested that in the *Mgat2*-deficient mice, fat uptake into the enterocytes was normal but lipids did not accumulate in the cells.

### Expression levels of genes involved in fatty acid oxidation in the small intestine and the liver

**Figure 7:**
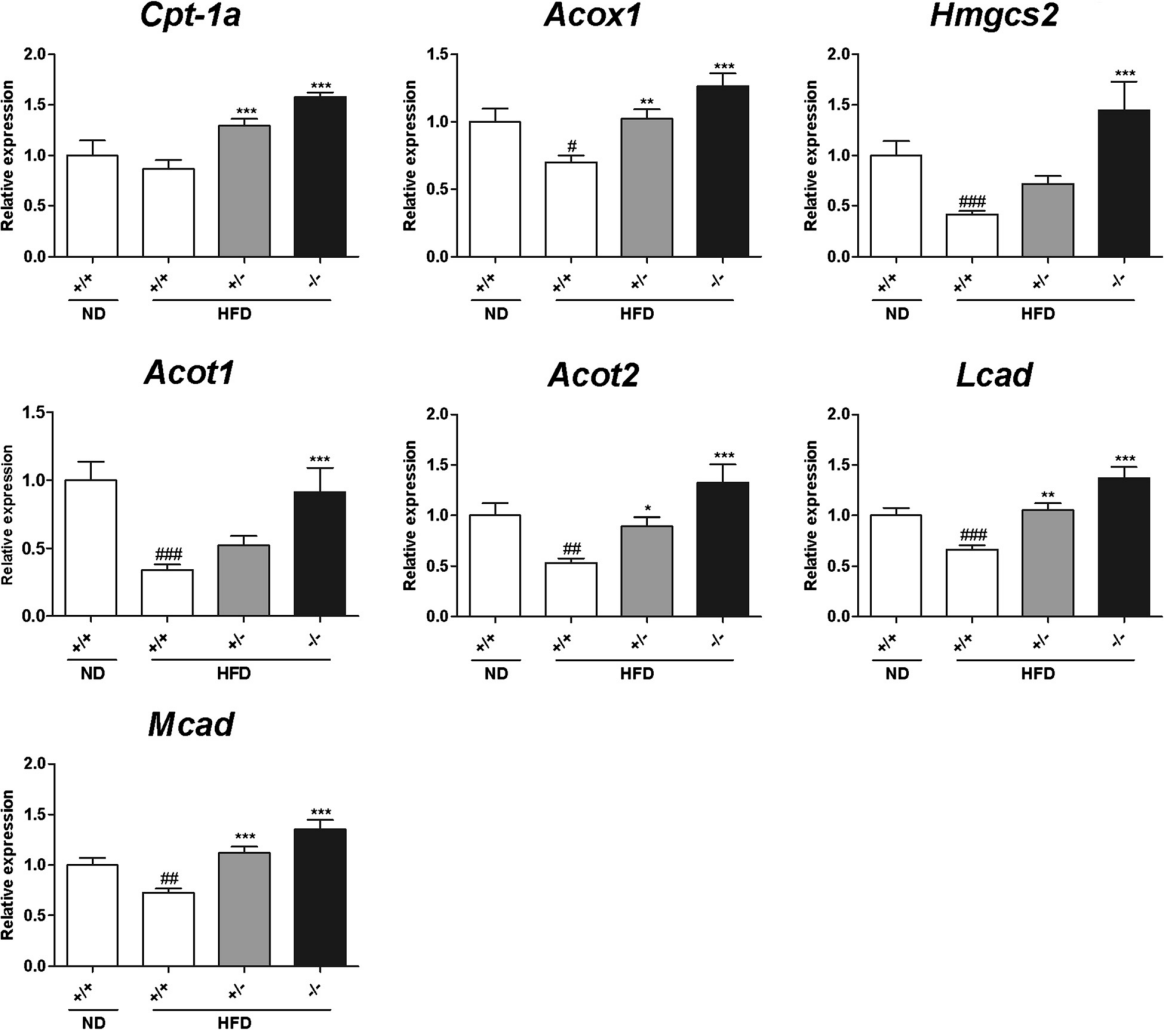
**Expression levels of genes involved in fatty acid oxidation in the duodenum of the*Mgat2*-deficient mice.** mRNA levels of genes involved in fatty acid oxidation were measured in the duodenum. Each bar represents mean ± SEM. n = 6–15 per group. ^*^P < 0.05, ^**^P < 0.01, ^***^P < 0.001 vs. HFD +/+. ^#^*P* < 0.05, ^##^*P* < 0.01, ^###^*P* < 0.001 vs. ND +/+.

**Figure 8:**
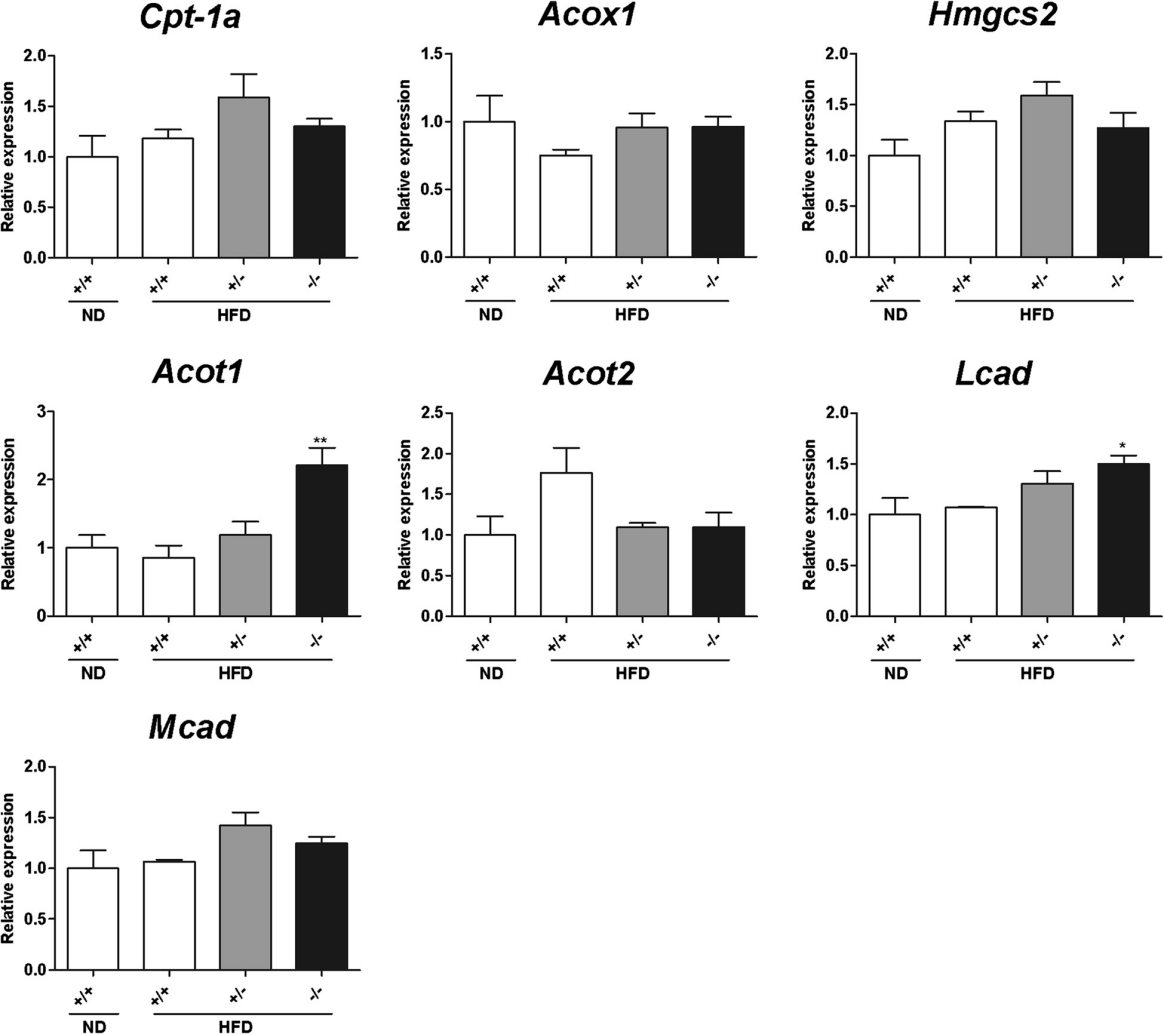
**Expression levels of genes involved in fatty acid oxidation in the liver of the*Mgat2*-deficient mice.** mRNA levels of genes involved in fatty acid oxidation were measured in the liver. Each bar represents mean ± SEM. n = 6–15 per group. ^*^P < 0.05, ^**^P < 0.01 vs. HFD +/+.

Considering the data obtained from our study, including that for reduced fat absorption into the circulation, normal fat uptake in enterocytes, and higher oxygen consumption in the *Mgat2*-deficient mice, we hypothesized that unabsorbed lipids were metabolized in the enterocytes of the *Mgat2*-deficient mice. Expression levels of genes involved in fatty acid oxidation, such as *Cpt-1a*, *Acox1*, *Hmgcs2, Acot1*, *Acot2*, *Mcad*, and *Lcad*, were significantly upregulated in the duodenum of the *Mgat2*-deficient mice (Figure 7). The increase of expression levels of these genes in the liver was not as obvious as that in the duodenum (Figure 8).

## Discussion

In this study, we constructed novel *Mgat2*-deficient mice and showed that these mice were protected from HFD-induced obesity and insulin resistance in accordance with a recent report by Yen *et al*. [12]. OFTT showed that fat absorption into the circulation was significantly reduced in the *Mgat2*-deficient mice as a result of inhibition of triglyceride resynthesis in the enterocytes. Decreased fat absorption may result in protection from obesity and insulin resistance in mice fed HFD for long-term. Improvement of insulin sensitivity might be explained not only by reduced weight gain but also by protection against lipotoxicity. Heterozygote (*Mgat2*^*+/−*^) mice had an intermediate phenotype between *Mgat2*^*+/+*^ and *Mgat2*^*−/−*^ and were partially protected from metabolic disorders, indicating a gene dosage-dependent effect of *Mgat2* and feasibility of MGAT2 as a potential drug target.

Regarding anti-obesity drugs, orlistat is a gastric and pancreatic lipase inhibitor that reduces dietary fat absorption [13]. However, orlistat has mechanism-based gastrointestinal adverse effects, including fatty and oily stool, fecal urgency, and fecal incontinence [13]. Fatty and oily stool was not observed in the *Mgat2*-deficient mice in our study. Fecal levels of triglycerides and NEFAs were comparable among genotypes. Taken together, the inhibition of MGAT2 may be a safer strategy than the inhibition of pancreatic lipase for the treatment of obesity.

Most of the triglycerides in food were absorbed into the enterocytes regardless of genotype because less than 1% of triglycerides were detected in the feces of any genotype. Reduced fat absorption into the circulation without obvious change in fat uptake into enterocytes suggested two distinct possibilities of lipid metabolism in the small intestine. One was the accumulation of lipids in the cells. However, triglyceride levels in the duodenum, jejunum, and ileum were comparable, and histological analysis also showed similar number and size of lipid droplets in the enterocytes among genotypes. Another possibility was immediate oxidation of lipids. The *Mgat2*-deficient mice exhibited higher oxygen consumption as reported previously [12,14]. Furthermore, expression levels of genes associated with fatty acid oxidation (*Cpt-1aAcox1Hmgcs2, Acot1Acot2Mcad*, and *Lcad*) were significantly upregulated in the duodenum of the *Mgat2*-deficient mice, whereas in the liver, increase of the gene expression levels was not as obvious as in the duodenum. Although the primary function of the small intestine is the resynthesis of triglycerides and the secretion of chylomicrons, it has been reported that the small intestine expresses oxidation-related enzymes, such as ACOX1 and MCAD, at levels comparable to those in the liver [15,16] and to a significant extent contributes to the metabolic rate and daily energy expenditure [17]. It is possible that activation of fatty acid oxidation in the enterocytes may lead to higher oxygen consumption in the *Mgat2*-deficient mice.

Genes involved in fatty acid oxidation were reported to have peroxisome proliferator-activated receptor (PPAR) response elements in their promoter region, and NEFAs are known to be endogenous ligands of PPARa [18-22]. Therefore, upregulation of these genes may be explained by the increased levels of NEFAs, substrates of MGAT2 enzyme, and endogenous ligands of PPARa, as a result of the inhibition of triglyceride resynthesis in the enterocytes. It should be stressed that this hypothesis requires further investigation including the enzyme activity of fatty acid oxidation in the small intestine.

Yen *et al.* previously reported that the mechanism by which *Mgat2* deficiency triggers oxygen consumption was unclear and mRNA expression of genes involved in fat oxidation in the small intestine and brown adipose tissue showed no differences among genotypes [12]. The difference in gene expression profiles in the small intestine between Yen’s study and our results could be explained by the measurement position used. We analyzed mRNA expression in the duodenum, whereas the jejunum was used in the study by Yen *et al*.

In mice, MGAT2 is mainly expressed in the small intestine. In contrast, in humans, MGAT2 is highly expressed both in the small intestine and liver[8,23]. It is well known that the hepatocytes play a key role in lipid metabolism by accumulating triglycerides and oxidizing fatty acid. Excessive storage and accumulation of lipid droplets into the hepatocytes result in hepatic steatosis. Therefore, the inhibition of MGAT2 activity might lead to lipid oxidation in the hepatocytes and improvement of fatty liver in humans.

## Conclusions

In summary, the *Mgat2*-deficient mice are protected from HFD-induced obesity and insulin resistance. MGAT2 has a pivotal role in lipid metabolism in the small intestine, and the inhibition of MGAT2 activity may be a promising strategy for the treatment of obesity and type 2 diabetes.

## Methods

### Generation of *Mgat2*-deficient mice

*Mgat2*-targeted 129/SvJae embryonic stem cells and, subsequently, mice carrying this mutation with a targeting vector designed to replace exon 1 of *Mgat2* with a neomycin-resistance cassette (Figure 1A) were generated. *Mgat2* deficiency was confirmed by Southern blotting of genomic DNA, which was digested with MfeI and SoeI using a probe hybridizing to sequences upstream of those included in the targeting vector, and by quantitative PCR. *Mgat2*^−/−^ and wild-type littermates were generated by breeding heterozygotes that were first backcrossed with C57BL/6 J mice. Mice were housed in a pathogen-free facility. Diets included a normal diet (ND, CRF-1, Oriental Yeast Co., 3.59 kcal g^−1^) and a high-fat diet (HFD, Oriental Yeast Co., 59% cal fat (lard), 5.578 kcal/g). Animals were maintained in a 12/12-h light–dark cycle. All experiments were performed on male animals. Experimental protocols concerning the use of laboratory animals were reviewed and endorsed by the Institutional Animal Care and Use Committee of Mitsubishi Tanabe Pharma Corporation.

### MGAT enzyme assays

MGAT activity was determined by measuring the incorporation of the [^14^ C]palmitoyl moiety into triacylglycerol with [^14^ C]palmitoyl-CoA (ARC, St. Louis, MO) and 2-monooleoylglycerol. Crude membranes (10 μg) of the small intestine were used as the enzyme source. Assays were performed in 200 μl buffer (100 mM Tris–HCl, pH 7.4, 200 mM sucrose, 5 mM MgCl_2_, 1.25 mg/ml BSA) containing 10 μM 2-monooleoylglycerol and 20 μM [^14^ C]palmitoyl-CoA. Reactions were performed for 5 min at 30°C and the products were extracted with 1 ml of chloroform:methanol (2:1, v/v). The extracts were dried and separated by thin layer chromatography with hexane:diethyl ether:acetic acid (80:20:1, v/v/v). Areas containing [^14^ C]triacylglycerol were visualized and quantitated using the FLA3000 fluorescence detection system (Fujifilm, Tokyo, Japan).

### Oral fat tolerance test (OFTT)

Mice were fasted overnight and then 10 ml/kg of Intralipos containing 20 % soybean oil (v/v) (Otsuka, Tokyo, Japan) was orally administered. Plasma samples were obtained from the tail vein before and 75, 150, 225, and 300 min after the fat challenge for determination of plasma triglyceride levels.

### Long-term feeding studies

Animals were weaned at three weeks of age and maintained on an ND or switched to an HFD at seven weeks of age. They were weighed and their food intake was monitored. After 10 weeks of HFD feeding, blood samples in the fed state and feces were collected for 3 days. After overnight fasting, the mice were killed by whole blood collection from the abdominal aorta under ether anesthesia. The small intestine and liver were quickly removed from each mouse, immediately frozen in liquid nitrogen, and stored at −80°C for quantitative PCR and lipid measurements.

### Determination of body composition

After 10–12 weeks of HFD feeding, the body composition was analyzed by dual-energy X-ray absorptiometry with PIXImus2 (GE Healthcare, Tokyo, Japan) under ether anesthesia.

### Determination of plasma parameters

Plasma triglyceride, total cholesterol, and non-esterified fatty acid (NEFA) levels were determined using an enzymatic assay kit (Wako Chemicals, Osaka, Japan). Blood glucose was determined using commercially available kits based on the glucose oxidase method (Wako Chemicals, Osaka, Japan). Plasma insulin was assayed using an enzyme-linked immunosorbent assay (ELISA) kit (Morinaga Co. Ltd., Yokohama, Japan).

### Oral glucose tolerance test (OGTT)

An oral glucose tolerance test (OGTT) was performed after 10 weeks of HFD feeding. Mice were fasted for 3 h and then 2 g/kg glucose solution was orally administered at a volume of 10 ml/kg. Blood samples were obtained from the tail vein before and 15, 30, 60, and 120 min after the glucose challenge for determination of blood glucose levels. Plasma samples were obtained before and 15, 30, and 60 min after the glucose challenge for determination of plasma insulin levels. HOMA-IR was calculated [24].

### Determination of lipid levels in feces and the small intestine

Fecal lipids were extracted with a 30-fold volume of chloroform:methanol (2:1, v/v) with vigorous shaking. Then, a 1/3.75-fold volume of water was added and the mixture was shaken vigorously. The organic phase was collected and again a 30-fold volume of chloroform:methanol (2:1, v/v) was added to the water phase. After vigorous shaking, the organic phase was collected, and the combined organic phase was evaporated. The dried residue was dissolved in isopropanol:Triton X-100 (9:1, v/v) and measured for triglyceride and NEFA levels using an enzymatic assay kit. The small intestine was homogenized in 20-fold volume of saline. Then, lipids were extracted with a 3.75-fold volume of chloroform:methanol (2:1, v/v) with vigorous shaking. The organic phase was collected and evaporated. The dried residue was dissolved in isopropanol:Triton X-100 (9:1, v/v) and measured for triglyceride levels using an enzymatic assay kit.

### Determination of oxygen consumption

After 10 weeks of HFD feeding, oxygen consumed by each mouse was measured using an ARCO-2000 analyzer (Arco System, Chiba, Japan) every 3 min for a 24-h period.

### Histology

Portions of duodenum were fixed in 10 % (v/v) neutral buffered formalin and embedded in paraffin. Paraffin sections (3–5 μm thick) were stained with hematoxylin and eosin and examined under a light microscope.

### Quantitative PCR

Total RNA of the duodenum and the liver was extracted without using DNase. Total RNA was reverse transcribed by TaqMan Reverse Transcriptase Reagents (Applied Biosystems, Foster City, CA). Real-time PCR was performed with SYBR Green PCR mix (Applied Biosystems, Foster City, CA) and analyzed with an ABI Prism 7000 Sequence Detection System (Applied Biosystems, Foster City, CA). The relative expression levels were compared after normalization to *B-actin*. The primers used for *B-actin* and *Mgat2* were from QIAGEN (Tokyo, Japan). The following primer pairs were used (5′ to 3′): *Cpt-1a*, forward: AAGCTGTTCAAGATAGCTTG, reverse: TGCTGATGACGGCTATGGTGT; *Acot1*, forward: GGCTGGGAATGGAGTTTCAT, reverse: GCTATCCAAGAAAAGTGCCAGG; *Acot2*, forward: AGTGCCTATGAAGGACTGAGGA, reverse: GGTAAAGGTGCTTTCTGCC; *Acox1*, forward: GGTAAAGGTGCTTTCTGCC, reverse: AGATAAACTCCCCAAGATTCAAGAC; *Hmgcs2*, forward: TGTCCCCTGAGGAATTCACAGAA, reverse: AACGAGTGGATGAGATGCATCG; *Lcad*, forward: CTGGTTAAGTGATCTCGTGATCGTCG, reverse: CTGGCAATCGGACATCTTCAAAGAATAGT; *Mcad*, forward GCTCTGATGTGGCGGCCATTA, reverse: AAGGCTTTACTAGCGGGTACTTTAGG

### Statistical analysis

All data were expressed as mean ± SEM. The comparison of mean values between two groups was performed by the Student’s t-test, and the Dunnett’s test was used for comparing more than two groups. *P* values of <0.05 were considered statistically significant.

## Abbreviations

MGAT, Acyl-coenzyme A:monoacylglycerol acyltransferase; DGAT, Acyl-coenzyme A:diacylglycerol acyltransferase; ND, Normal diet; HFD, High-fat diet; OFTT, Oral fat tolerance test; OGTT, Oral glucose tolerance test; HOMA-IR, Homeostasis model assessment for insulin resistance; NEFA, Non-esterified fatty acid; ELISA, Enzyme-linked immunosorbent assay; Cpt-1a, Carnitine palmitoyltransferase-1a; Acox1, Acyl-coenzyme A oxidase 1; Hmgcs2, 3-hydroxy-3-methyl-glutaryl-coenzyme A synthase 2; Acot, Acyl-coenzyme A thioesterase; Mcad, Medium-chain acyl dehydrogenase; Lcad, Long-chain acyl dehydrogenase; PPAR, Peroxisome proliferator-activated receptor.

## Competing interests

The authors declare that they have no competing interests. The authors alone are responsible for the content and writing of the paper.

## Authors’ contributions

TT designed the study, carried out the animal experiments and quantitative PCR, and drafted the manuscript. SF, NT, TI carried out the animal experiments. HA and KW carried out the generation of Mgat2-deficient mice. HS carried out the histological analysis. NO and MS participated in the design of the study. AO conceived of the study, and participated in its design and coordination and helped to draft the manuscript. All authors read and approved the final manuscript.
